# Clinical Performance of Partial and Full-Coverage Fixed Dental Restorations Fabricated from Hybrid Polymer and Ceramic CAD/CAM Materials: A Systematic Review and Meta-Analysis

**DOI:** 10.3390/jcm9072107

**Published:** 2020-07-04

**Authors:** Nadin Al-Haj Husain, Mutlu Özcan, Pedro Molinero-Mourelle, Tim Joda

**Affiliations:** 1 Department of Reconstructive Dentistry and Gerodontology, School of Dental Medicine, University of Bern, 3010 Bern, Switzerland; pedro.molineromourelle@zmk.unibe.ch; 2 Division of Dental Biomaterials, Clinic for Reconstructive Dentistry, Center for Dental and Oral Medicine, University of Zurich, 8032 Zurich, Switzerland; mutlu.ozcan@zzm.uzh.ch; 3 Department of Reconstructive Dentistry, University Center for Dental Medicine Basel, University of Basel, 4058 Basel, Switzerland; tim.joda@unibas.ch

**Keywords:** bonding, CAD/CAM, composite resin cement, dental, hybrid polymer, indirect, meta-analysis, systematic review

## Abstract

The aim of this systematic review and meta-analysis was to evaluate the clinical performance of tooth-borne partial and full-coverage fixed dental prosthesis fabricated using hybrid polymer and ceramic CAD/CAM materials regarding their biologic, technical and esthetical outcomes. PICOS search strategy was applied using MEDLINE and were searched for RCTs and case control studies by two reviewers using MeSH Terms. Bias risk was evaluated using the Cochrane collaboration tool and Newcastle–Ottawa assessment scale. A meta-analysis was conducted to calculate the mean long-term survival difference of both materials at two different periods (≤24, ≥36 months(m)). Mean differences in biologic, technical and esthetical complications of partial vs. full crown reconstructions were analyzed using software package R (*p* < 0.05). 28 studies included in the systematic review and 25 studies in the meta-analysis. The overall survival rate was 99% (0.95–1.00, ≤24 m) and dropped to 95% (0.87–0.98, ≥36 m), while the overall success ratio was 88% (0.54–0.98; ≤24 m) vs. 77% (0.62–0.88; ≥36 m). No significance, neither for the follow-up time points, nor for biologic, technical and esthetical (88% vs. 77%; 90% vs. 74%; 96% vs. 95%) outcomes was overserved. A significance was found for the technical/clinical performance between full 93% (0.88–0.96) and partial 64% (0.34–0.86) crowns. The biologic success rate of partial crowns with 69% (0.42–0.87) was lower, but not significant compared to 91% (0.79–0.97) of full crowns. The esthetical success rate of partial crowns with 90% (0.65–0.98) was lower, but not significant compared to 99% (0.92–1.00) of full crowns.

## 1. Introduction

Over the past two decades, metal-free computer-aided design/computer aided manufacturing (CAD/CAM) materials, including ceramics and composites, have been widely used in dentistry [1]. In the restorative clinical field, these materials have been gaining importance due to their biologic and esthetical properties resulting in favorable treatment outcomes in order to satisfy increased demands and expectations of patients and dentists [2,3].

The improvements in oral health during the last decades, have promoted less aggressive dental preparations changing the conventional indications and workflows of these restorations and adapting it for these metal-free materials [4,5]. The current state of the art of dental treatments accompanied by life changes in terms of time efficacy and patient care demands, have fostered the introduction of faster and cost-efficient digital clinical workflows using CAD/CAM technology facilitating high quality restorative treatments [6,7]. These workflows allow designing and manufacturing of chairside partial or full-contoured monolithic restorations, such as inlays, veneers, single crowns (SCs) or multi-spans fixed dental prostheses (FDPs), with esthetically favorable appearance, accurate marginal adaptation in a cost and time efficient production manner [3,8].

Digital technologies also enabled the development of high-performance materials like Lithium disilicate (LD), Lithium aluminosilicate ceramic reinforced with lithium disilicate glass–ceramic (LD-LAS), hybrid-polymer ceramic (HPC) and resin-matrix ceramics (RMC) including resin-based ceramics (RBC) and polymer infiltrated ceramic network (PICN) resins [9,10,11].

LD is one of the of the most commonly used chairside material due to its great clinical performance and high acceptance by patients, technicians and dentists. LD-LAS covers the same indication range as LD ceramics, while showing comparable flexural strength tests results, making it a high load-bearing material with excellent esthetic properties [12,13]. The group of hybrid materials (HPC, RMC, RBC and PICN) are of growing interest due their mechanical resistibility and high elasticity. These materials are based on a ceramic like hybrid ceramic also known as resin-matrix-ceramics, resin-based ceramics or nanoceramics, presenting promising results, as they follow esthetic trends combined with minimally invasive preparations in modern clinical workflows [11,14].

The gold standard in SCs and FDPs is still ceramic fused to metal. This “conventional” approach often presents esthetic shortcomings, requires a more aggressive tooth preparation and extended technical production time. Therefore, metal-free options have gradually become a favorite alternative compared to metal-ceramic restorations [15,16]. However, when using metal-free materials, clinicians should keep in mind the limited evidence that these materials present in terms of long-term performance, survival and complication rates and carefully evaluate the indication and processing technique in each unique clinical case [14].

The wide range of new hybrid polymer and ceramic CAD/CAM materials that are offered in the dental industry to manufacture tooth-borne restorations implies the need for an evidence-based study that evaluates the current clinical behavior of these materials. Therefore, the aim of this systematic review and meta-analysis was to analyze the clinical behavior of partial and full fixed restorations out of hybrid polymer and ceramic CAD/CAM materials. This present systematic review was performed in order to answer the PICO question defined as follows: In patients receiving tooth-borne partial or full crowns, are survival and clinical success rates of monolithic CAD/CAM restorations comparable to those of conventionally manufactured?

## 2. Experimental Section

### 2.1. Search Strategy

A preliminary search was conducted prior to the definition of the final PICO question, focusing on material choice (glass ceramic multiphase (e.g., Enamic); polymeric multiphase (e.g., Lava Ultimate)); Indication (tooth and implant-borne single-unit restoration and reconstruction design (crown vs. partial crown single unit).

The PICO question was then chosen as follows: *P-*population: tooth-borne partial or full crowns; I-intervention: Monolithic CAD/CAM restorations; C-control: conventionally produced/manufactured restorations (natural teeth); O-outcome: survival and clinical success (fracture, debonding, behavior); S-study designs: randomized control trials (RCT) and case–control studies.

The following MeSH terms, search terms and their combinations were used in the PubMed search: ((((((((dental crowns [MeSH]) OR (dental restoration permanent [MeSH]) OR (full crown) OR (partial crown) OR (table top))))) AND ((((computer-aided design [MeSH])) OR (computer-assisted design [MeSH]) OR ((computer-aided manufacturing [MeSH])) OR (computer-assisted manufacturing [MeSH]) OR (cerec [MeSH]) OR (CAD/CAM) OR (rapid prototyping))))) OR ((((ceramics [MeSH]) OR (dental porcelain [MeSH]) OR (polymers [MeSH]) OR (monolithic))))) AND ((((survival analysis [MeSH terms]) OR (survival rate [MeSH Terms]) OR (survival))))) OR ((((success) OR (failure) OR (dental restoration failure [MeSH terms]) OR (complications [MeSH terms]) OR (clinical behavior) OR (adverse event) OR (chipping) OR (debonding)))). The search strategy according to the focused PICOS question is presented in Table 1.

The following terms were used in the EMBASE search: (‘dental crowns’/exp OR ‘dental restoration permanen’/exp OR ‘full crown’/exp OR ‘partial crown’/exp OR ‘table top’) AND (‘ computer-aided design’ OR ‘computer-assisted design’ OR ‘computer-aided manufacturing’ OR ‘ computer-assisted manufacturing’ OR ‘cerec’ OR ‘CAD/CAM’ OR ‘rapid prototyping’) OR (‘ceramics’ OR ‘dental porcelain’ OR ‘polymers’ OR ‘monolithic’) AND (‘survival analysis’ OR ‘survival rate’ OR ‘survival’) OR (‘success’ OR ‘failure’ OR ‘dental restoration failure’ OR ‘complications’ OR ‘clinical behavior’ OR ‘adverse event’ OR ‘chipping’ OR ‘debonding’) NOT [medline]/lim AND [embase]/lim.

The following terms were used in the Web of Science and IADR abstracts search: ((((((((dental crowns [MeSH]) OR (dental restoration permanent [MeSH]) OR (full crown) OR (partial crown) OR (table top))))) AND ((((computer-aided design [MeSH])) OR (computer-assisted design [MeSH]) OR ((computer-aided manufacturing [MeSH])) OR (computer-assisted manufacturing [MeSH]) OR (cerec [MeSH]) OR (CAD/CAM) OR (rapid prototyping))))) OR ((((ceramics [MeSH]) OR (dental porcelain [MeSH]) OR (polymers [MeSH]) OR (monolithic))))) AND ((((survival analysis [MeSH Terms]) OR (survival rate [MeSH Terms]) OR (survival))))) OR ((((success) OR (failure) OR (dental restoration failure [MeSH Terms]) OR (complications [MeSH Terms]) OR (clinical behavior) OR (adverse event) OR (chipping) OR (debonding)))).

### 2.2. Information Sources

A systematic electronic literature search was conducted in PubMed MEDLINE, EMBASE and Web of Science (ISI—Web of Knowledge), including Google Scholar and IADR abstracts until 16 May 2018. The search aimed for English language clinical trials and case–control studies published in the last five years, performed on human and published in dental journals. Search syntax was categorized in a population, intervention, comparison and outcome study design; each category assembled using a combination of Medical Subject Heading [MeSH Terms].

### 2.3. Study Selection and Eligibility Criteria

To minimize the potential for reviewer bias, two reviewers (N.A.-H.H. and T.J.) independently conducted electronic literature searches and the study selection. Both reviewers studied the retrieved titles and abstracts and disagreements were solved by discussion. Forty-eight selected studies were then obtained in full texts, and the decision of inclusion of studies was made according to preset inclusion criteria.

The following inclusion criteria were chosen for the articles included in this systematic review: (1) RCTs and case control studies; (2) Studies with observation of a follow-up period of ≥1 year; (3) Studies that considered either hybrid polymers or ceramic CAD/CAM materials.

**Figure 1:**
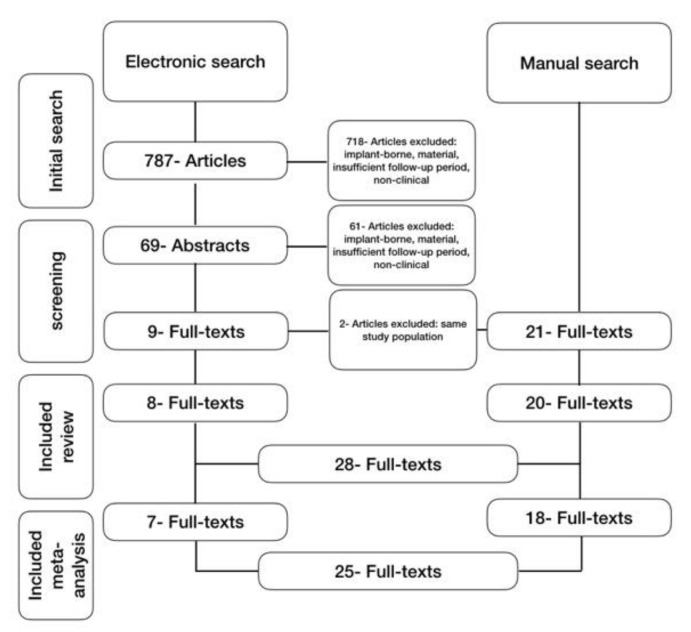
Flow diagram of the systematic search results.

Articles meeting one or more of the following criteria were excluded: (1) In vitro or in situ studies; (2) Studies with a follow-up period less than one year; (3) Studies testing materials other than hybrid polymers or ceramic CAD/CAM materials. For quantitative analyses (meta-analysis), studies lacking a control group or standard deviation values were excluded (Figure 1).

### 2.4. Data Extraction and Collection

After screening the data, extracting, obtaining and screening the titles and abstracts for inclusion criteria, the selected abstracts were obtained in full texts. Titles and abstracts lacking sufficient information regarding inclusion criteria were also obtained as full texts.

Full text articles were selected in case of compliance with inclusion criteria by the two reviewers using a data extraction form. Two reviewers (N.A.-H.H. and T.J.) independently collected the following data from the included articles for further analysis: demographic information (title, authors, journal and year), study specific parameter (study type, number of treated patients, number of restorations, Ratio (restorations/patient), follow-up and drop-out), materials tested (type and commercial name, manufacturing process, luting agent, failure, survival and success rate), means and standard deviations of the clinical parameters (biologic, technical and esthetical failures).

The authors of the studies were contacted in case of unpublished data. These studies were only included if the authors provided the missing information. In order to assess the clinical performance and outcomes of the restorations, the selected studies based their evaluations on the modified United States Public Health service (USHPS) [17] criteria and the FDI World dental federation criteria [18].

For the extraction of the clinical outcomes, the relevant data of the included studies were divided into three subgroups according to their evaluated outcomes, based on the USHPS criteria and the FDI criteria: The USHPS criteria are based on an evaluation of the clinical characteristics of color, marginal adaptation, anatomic form, surface roughness, marginal staining, secondary caries and luster of restoration which is evaluated on three levels form the best to worst outcome, Alpha, Bravo and Charlie.

The FDI criteria are based on three levels that were scored into five points (Clinically very good, clinically good, clinically sufficient/satisfactory, clinically unsatisfactory, clinically poor): (A) Esthetic properties that evaluate the surface luster, the staining, color match and translucency and the esthetic anatomic form; (B) Functional properties based on the assess of fracture of material and retention, the marginal adaptation, the occlusal contour and wear, the approximal anatomic form, the radiographic examination and the patient’s view; (C) Biologic properties measure the postoperative sensitivity and tooth vitality, the recurrence, the tooth integrity of caries, the periodontal response, the adjacent mucosa and the oral and general health.

### 2.5. Risk of Bias Assessment

The risk of bias assessment was evaluated using the Cochrane collaboration tool for randomized studies, evaluating bias risks such as sample size calculation, random sequence generation, adequate control group, materials usage following the manufacturers’ instructions, tests execution by a single blinded operator, adequate statistical analysis, allocation concealment, completeness of outcome data, selective reporting and other bias. Each parameter reported by the included studies was recorded. Articles that included only one to three possible risks of bias of these items were considered at low risk for bias; four or five items, at medium risk for bias; and six to nine items, at high risk for bias.

In case of a high or unclear risk of bias the study was assigned to a judgment of risk of bias. The Newcastle–Ottawa assessment scale was applied for non-randomized studies, for the selection of the study groups, the comparability of the groups and the ascertainment of outcome or interest.

### 2.6. Data Analyses

The statistical analysis was performed with the software package R, Version 3.5.3 (R Core Team 2013) [19]. Both survival and success ratios were analyzed performing a meta-analysis using the logit transformation method. Results of the random effects model were reported and forest plots were drawn. Funnel plots were also produced in order to detect a possible publication bias. Overall, survival and success ratios were analyzed as well as biologic, technical and esthetical successes. The restorations instead of patients were used as the statistical unit. Studies that lacked the required information of the sample size or the follow-up time were excluded from the statistical analysis. All materials had to be pooled because of sample size considerations or missing information. The meta-analysis was done with studies reporting a follow-up time of at least 24 months.

## 3. Results

### 3.1. Study Selection

Of 795 potentially relevant studies, 48 were selected for a full-text analysis, 28 were included in the systematic review and 25 considered in the meta-analysis. Eight full text articles were selected using electronic databases and 20 further were retrieved throughout manual search. From the 25 studies included in the meta-analysis, 12 studies were randomized controlled trial, 14 prospective and 2 retrospectives (Krejci et al. 1992; Taskonak et al. 2006; Frankenberger et al. 2008; Frankenberger et al. 2009; Dukic et al. 2010; Fasbinder et al. 2010; Manhart et al. 2010; Azevedo et al. 2012; Esuivel-Opshaw et al. 2012; Murgueitio et al. 2012; Schenke et al. 2012; Taschner et al. 2012; Gehrt et al. 2013; Reich et al. 2013; Akin et al. 2014; D’all’Orologio et al. 2014; Dhima et al. 2014; Guess et al. 2014; Guess et al. 2014; Selz et al. 2014; Seydler et al. 2015; Baader et al. 2016; Botto et al. 2016; Mittal et al. 2016; Özsoy et al. 2016; Santos et al. 2016; Rauch et al. 2018) [20,21,22,23,24,25,26,27,28,29,30,31,32,33,34,35,36,37,38,39,40,41,42,43,44,45,46].

### 3.2. Study Characteristics

The characteristics of the included studies are presented in Table 2. The included articles were published between 1992 and 2018. A total of type of 28 studies including 1150 patients and 2335 reconstructions with a mean follow-up time of 4.5 years (min–max: 1–18 years) were evaluated. Materials included were composites, feldspathic ceramic, leucite reinforced glass ceramic, veneered and non-veneered lithium disilicate, veneered and monolithic zirconia and alumina. Processing techniques were stone dies incremental techniques and poured with dental stone, indirect die cast method, framework laminated with a veneering with lost-wax glaze technique, chairside and labside CAD/CAM techniques, vacuum injection mold techniques. Used luting agents were adhesive bonding systems, resin cements (Panavia, Multilink, Variolink, Tetric, Multibond) and glass ionomer luting cements (Ketac).

### 3.3. Risks of Bias in Individual Studies

**Figure 2:**
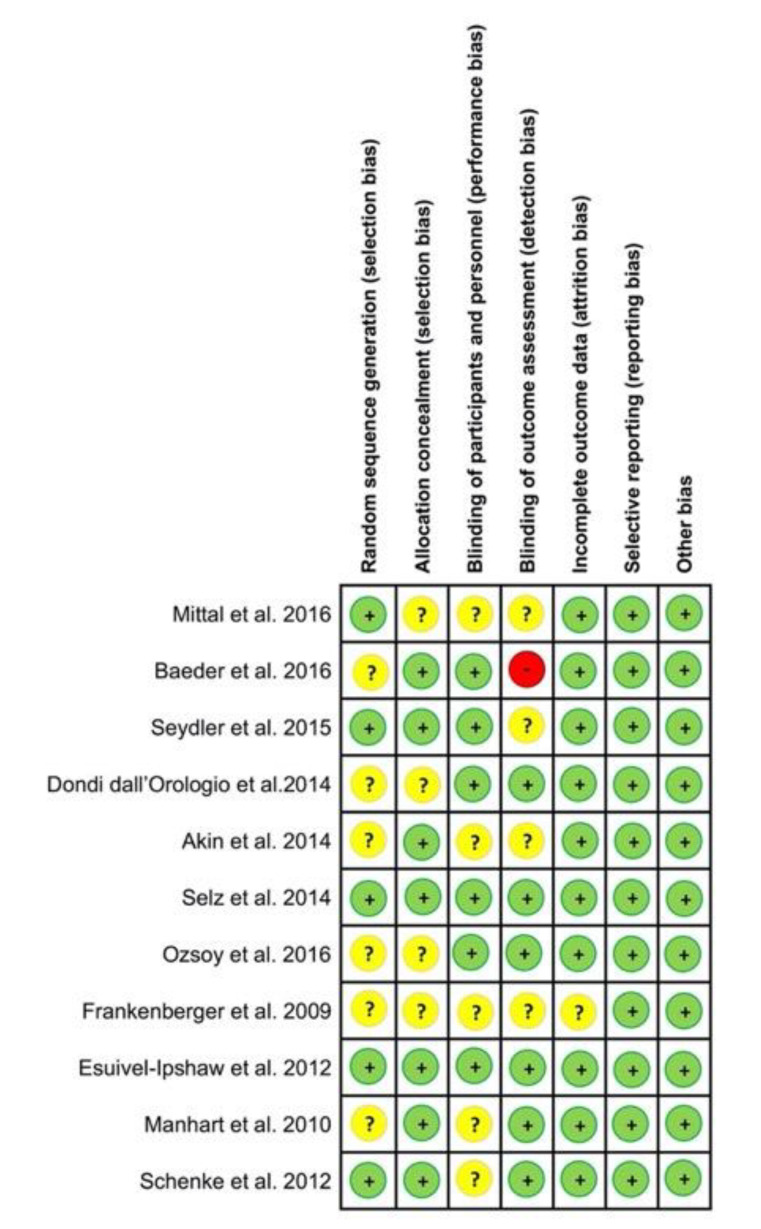
Summary of the Cochrane collaboration tool for assessing risk of bias for randomized controlled trials.

Quality and risk bias assessment of the RCTs is summarized in Figure 2 and for the case control and cohort studies reviewed in Table 1.

The Cochrane collaboration tool showed an overall low risk of bias in all the included studies. Some studies did not report enough information about the sequence generation process to allow an evaluation of either “low risk” or “high risk” (Mittal et al. 2016, Frankenberger et al. 2009). Others did not describe the allocation concealment or provide enough detail (Mittal et al. 2016, Dondi dall’Orologio et al. 2014, Ozsoy et al. 2016, Frankenberger et al. 2009). Just one study showed a high risk for the blinded outcome (Beder et al. 2016). According to the NOS scale, one study scored 2 points, two obtained 3 points, two 4 points, one 5 points, and finally seven studies obtained 8 points. These scores reflect an adequate quality of the studies included in this review.

### 3.4. Meta-Analysis

Meta-analyses were performed based on 25 studies. The overall survival and success ratios of partial and full crowns were obtained using forest and funnel plots at two different time ranges: (a) ≤24 months (m); and (b) ≥36 months (m) (Table 3).

### 3.5. Survival Ratios

**Figure 3:**
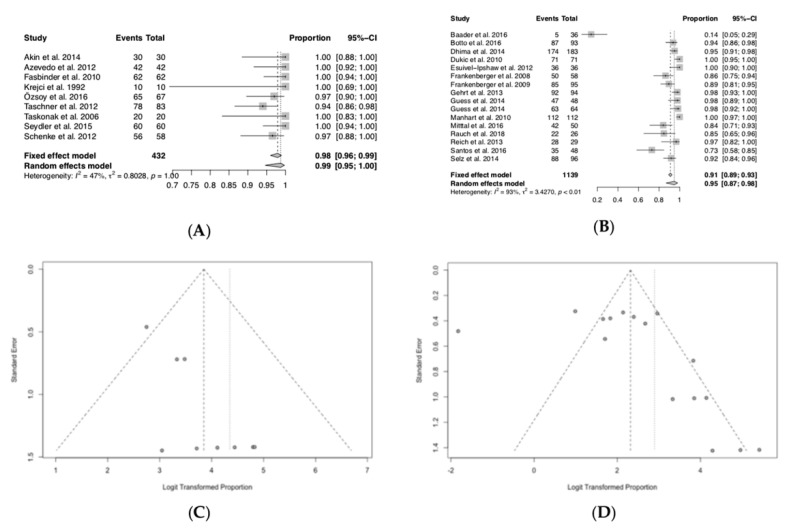
Survival ratios of all included specimens. (**A**) Forest plot ≤24 months; (**B**) forest plot ≥36 months; (**C**) funnel plot ≤24 months; (**D**) funnel plot ≥36 months.

**Figure 4:**
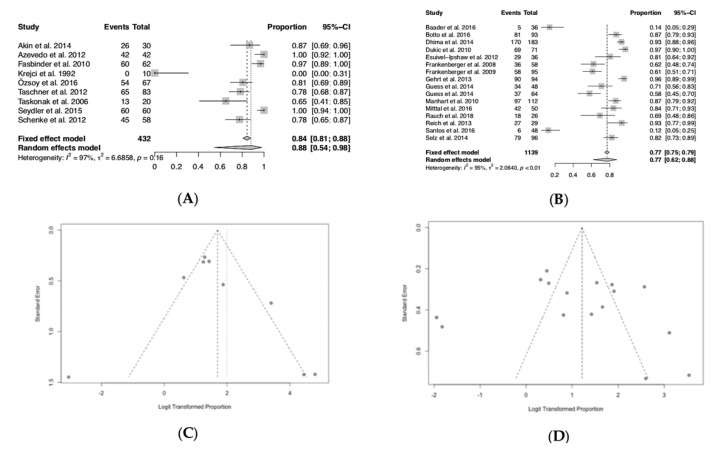
Success ratios of all biologic, technical and esthetical aspects. (**A**) Forest plot ≤24 months; (**B**) forest plot ≥36 months; (**C**) funnel plot ≤24 months; (**D**) funnel plot ≥36 months.

**Figure 5:**
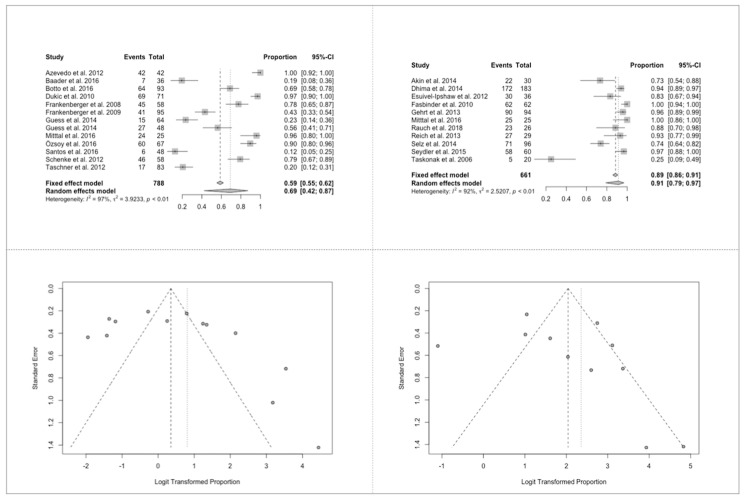
Success ratios of all biologic aspects. (**A**) Forest plot for partial and (**B**) full crowns; (**C**) funnel plot for partial and (**D**) full crowns.

**Figure 6:**
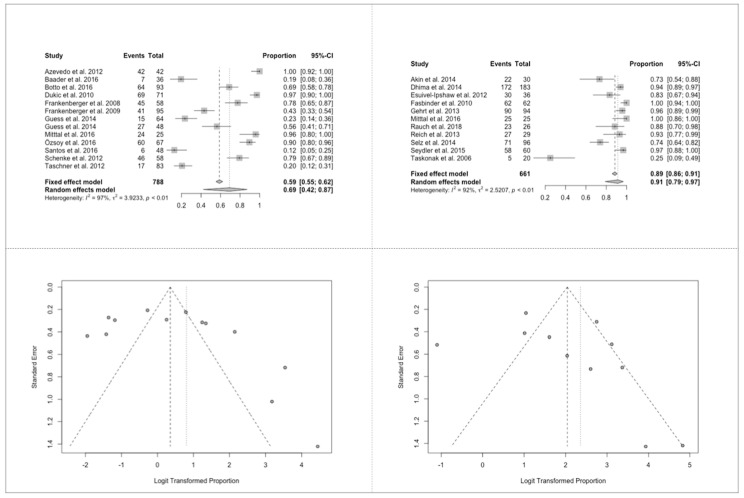
Success ratios of all technical aspects. (**A**) Forest plot for partial and (**B**) full crowns; (**C**) funnel plot for partial and (**D**) full crowns.

**Figure 7:**
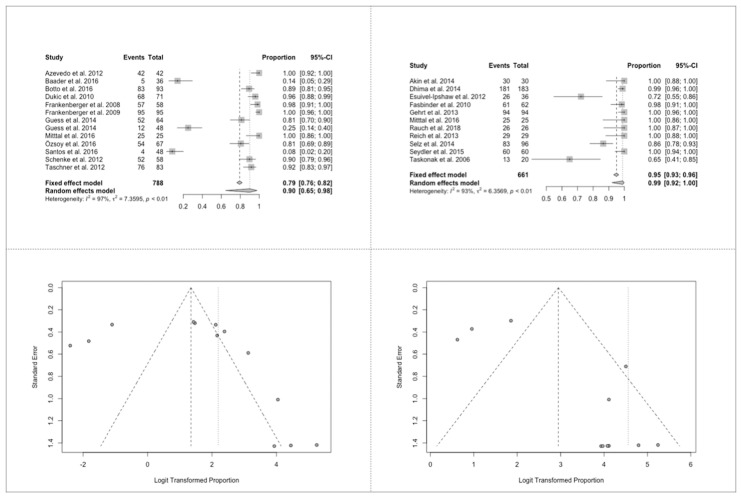
Success ratios of all esthetical aspects. (**A**) Forest plot for partial and (**B**) full crowns; (**C**) funnel plot for partial and (**D**) full crowns.

As for the survival ratios it could be observed that at the time frame up to 24 m the estimated survival is 99%, while after at least 36 m it dropped to 95%. Forest and funnel plots ≤24 m revealed homogeneous results (heterogeneity $I^{2}$ = 47%, *p* = 1.00) and low suspicion for a publication bias, while forest and funnel plots ≥36 m demonstrated heterogeneous results (heterogeneity $I^{2}$ = 93%, *p* < 0.01) and a slight suspicion of a publication bias (Figure 3, Figure 4, Figure 5, Figure 6 and Figure 7).

### 3.6. Success Ratios of All Biologic, Technical and Esthetical Aspects

The estimated success ratio at ≤24 m was 88% (95% COI: 0.54–0.98), while after at least 36 m it dropped to 77% (95% COI: 0.62–0.88). Forest plot ≤24 m revealed not strongly homogeneous results (heterogeneity $I^{2}$ = 97%, *p* = 0.16). However, heterogeneity is not statistically significant. Funnel plot ≤24 m showed very small and extremely large values. Forest plot ≥36 m demonstrated highly heterogeneous results ($I^{2}$ = 95%, *p* < 0.01). The plot illustrates the studies with the remarkably noticeable results. The wide range and heterogeneity of included material types (composites, feldspathic ceramic, leucite reinforced glass ceramic, veneered and non-veneered lithium disilicate, veneered and monolithic zirconia and alumina), processing techniques and luting agents did not allow any further statistical analysis as regards to an analysis for the material type only.

### 3.7. Success Ratios of All Biologic Criteria

The estimated success ratio at ≤24 m was 88% (95% COI: 0.58–0.97), while after at least 36 m it dropped to 75% (95% COI: 0.56–0.88). Results of the forest Plot <24 m presented very heterogeneous results ($I^{2}$ = 96%, *p* < 0.01). The funnel Plot <24 m showed, apart from the before mentioned two studies the distribution of published results, a slight skew in favor of high success rates, indicating a possible publication bias.

For forest plot >36 m ($I^{2}$ of 97%, *p* < 0.01) these study results were also very heterogeneous, and a large dispersion could be observed. In general, the results of the funnel Plot >36 m presented great variability among the published studies.

### 3.8. Success Ratios of All Technical Criteria

After 2 years the estimated success ratio was 90% (95% COI: 0.74–0.97), while after 3 years it dropped to 74% (95% COI: 0.50–0.89). Forest plot <24 m presented ($I^{2}$ of 93%, *p* < 0.01) heterogenous results and after 3 years ($I^{2}$ of 97%, *p* < 0.01). The funnel plot after 2 years showed a tendency towards overproportioned high success rates studies.

### 3.9. Success Ratios of All Esthetical Criteria

The success ratios are very high at 24 m 96% (95% COI: 0.87–0.99) and dropped very slightly after 36 m 95% (95% COI: 0.78–0.99). Forest plot <24 m presented ($I^{2}$ of 86%, *p* = 0.08) statistically insignificant heterogenous results and after 3 years ($I^{2}$ of 97%, *p* < 0.01) heterogenous results, because of 3 studies showing only 8%–25% success rates, while all other included studies presented ≥72%. Funnel plot did not show any bias during the first 2 years, while the 3 mentioned studies presented very low success rates, many others shower too high success rates. The overall results did not show any bias.

The biologic success rates of full crowns were much higher than those of partial crowns. Forest plot of partial ($I^{2}$ of 97%, *p* < 0.01) and full ($I^{2}$ of 92%, *p* < 0.01) crowns showed very heterogeneous studies, while funnel plots exhibited a possibility of publication bias for partial and low possibility of bias for full crowns, even though there was a slight hint of too high success rates.

The technical success rates of full crowns were much higher and significantly different (*p* < 0.05) compared to partial crowns. Forest plot showed heterogeneous results for partial crowns ($I^{2}$ of 98%, *p* < 0.01) and homogeneous results for full crowns ($I^{2}$ of 66%, *p* = 0.63). Funnel plot for partial crowns showed a rather unlikely publication bias, the variation is very high, for full crowns the results were all in the expected range, with an asymmetric distribution. Higher success rates were often demonstrated as statistically expected. A publication bias seems to be possible.

The esthetical success of partial crowns was also higher compared to full crowns, but not as high as it was for biologic and technical success rates. Forest plot of partial crowns ($I^{2}$ of 97%, *p* < 0.01) revealed heterogeneous results with three studies showing low success rates, the funnel plot exhibited at both sides a high prevalence of studies in the upper and lower end of the graph with more studies presenting high results. The forest plot of full crowns ($I^{2}$ of 93%, *p* < 0.01) showed also heterogeneous results, because of the two studies Esquivel-Ipshaw et al. and Taskonak et al. reporting low results. The funnel plot showed many results with high success rates and three with low results. Because of the sample size it was not possible to conclude if a bias was possible or not.

## 4. Discussion

This systematic review including meta-analysis was conducted to evaluate the clinical short- and long-term survival rates and biologic, technical and esthetical success ratios of partial and full crowns using hybrid polymer and ceramic CAD/CAM materials.

Some data were reported on CAD/CAM processing methods regarding survival and clinical survival rates. However, to best of author’s knowledge, no similar systematic review based on hybrid polymer and ceramic materials on survival and complications rates has been published yet. Since these materials have been developed recently, their indications and clinical applicability are still being studied. In the present review, the existence of a great variety and heterogeneity of hybrid polymer and ceramic materials and their indications has been observed.

The meta-analysis of this study was performed for mean long-term survival rates and for biologic, technical and esthetic complication ratios for partial vs. full crown reconstructions at two different follow-up periods. Due to the variety of the CAD/CAM materials, their differing compositions and the lack of homogeneity, the variable “material” could not be included in the meta-analysis. This finding was also observed in the systematic review by Alves de Carvalho et al. [47]. investigating clinical survival rates in single restorations using CAD/CAM technologies with a minimum follow-up of three years, describing a great variety of studies analyzing different materials. Their results are in agreement with the present systematic review related to the heterogeneity caused by the variety of the materials assessed [47]. The review of Rodrigues et al. included studies on CAD/CAM materials for single crown, multiple- unit or partial ceramic crown with a 24 to 84-month follow-up based on the longevity and failures rates, suggesting that the longevity of CAD/CAM restorations is lower compared to the conventionally fabricated restorations [48], as they presented a 1.84 higher failure rate during a follow-up period of 24 to 84 months. However, the results of the present systematic review showed that when partial and full crown reconstructions made of hybrid polymer and ceramic CAD/CAM materials were analyzed, the overall survival rate was 99% (0.95–1.00) up to 24 months and dropped to 95% (0.87–0.98) at ≥36 months.

These results were assessed based on the restoration type, given higher success rates for the overall clinical performance in full crown reconstructions compared to partial crowns. Similar data were found for survival rates of full crowns, estimated 5-year survival rate for leucite or lithium-disilicate reinforced glass ceramic (96.6%) and sintered alumina and zirconia (96%) were similar [16]. For partial restorations, our results are also in agreement with the literature, Sampaio FBWR et al. found estimated survival rates for CAD/CAM of 97% after five years [49].

Current trends for material selection in tooth-supported single restorations showed that, both clinicians and patients are favoring esthetic and nonmetallic restorations. However, for full crowns, literature is still supporting the porcelain-fused-to-metal crowns as the gold standard, with results of 5-year survival rates exceeding 95% [16,50]. Furthermore, in terms of longevity, the literature showed that full and partial CAD/CAM ceramic crowns have lower long-term survival compared to the ones produced through conventional techniques [48]. Analyzing the results of other studies of full ceramic crowns, the literature provided data on leucite or disilicate reinforced ceramics survival rates of 96.6% and 95%, respectively [16], these results are comparable to those found in this review.

The other large CAD/CAM processed material group was zirconia, showing a 5-year survival of 91.2% (82.8–95.6%) [16]. Digital developments, new materials and advanced processing techniques enabled the minimal invasive approach in dentistry throughout partial restorations. Partial crowns have been widely used for years, as composite resins were a less predictable treatment option for direct restorations. Among other factors, the longevity of partial restorations depended on the restorative material, the patient and the experience of the clinician. Previous reviews show survival rates of 92% and 95% at five years and 91% at 10 years, (Morimoto et al.) or in a more recent study the survival rate data for inlays was 90.89% and 93.50% in a follow-up period of one to five years [51].

Gold alloys have served as gold standard for partial crowns for years [52]. However, the increasing price of gold and the high esthetic demands of patients have caused advancement of materials such as hybrid polymer and ceramic CAD/CAM materials. The current evidence of gold restorations is limited, suggesting a survival rate of 95.4% observed in a retrospective, clinical study studying 1314 gold restorations; whereas inlays had a failure rate of 4.7% after more than 20 years [53]. Another study evaluated 391 posterior gold inlays during a mean follow-up period of 11.6 years and observed 82.9% of success rate and a 6.4% failure rate [52].

The development, evolution and improvement of composite resins, high strength ceramics and adhesive techniques have allowed the development of hybrid materials to compensate the deficiencies and limitations of gold alloys. In this regard, a systematic review evaluating 5811 restorations showed a survival rate of feldspathic porcelain and glass–ceramics for five-year follow-up of 95% and at the 10-year follow-up of 2154 restorations, a survival rate of was 91% [54].

In addition to ceramics and gold alloys composite resin materials have been increasingly used due to improvements in the composition and thereby related mechanical properties. Previous reviews on resins were inconclusive whether longevity and survival rates of resins are higher compared to ceramics [55]. However, a recent review on CAD/CAM materials for full and partial crowns that included resin-matrix ceramic showed an estimated survival rate after five years of 82.5% [47,49].

Survival rates are a reliable indicator to assess clinical performance. However, after placement and during exposure to the oral cavity restorations can present complications compromising their longevity, survival and clinical success. The clinical performance based on the overall success ratio of biologic, technical and esthetical aspects was 88% (0.54–0.98; ≤24 m) vs. 77% (0.62–0.88; ≥36 m) for the different follow-up periods. The meta-analysis could not find any significance regarding both follow-up time (≤24 m or ≥36 m) and their biologic, technical and esthetical (88% vs. 77%; 90% vs. 74%; 96% vs. 95%) outcome. However, it presented a significant difference in the technical clinical performance between full 93% (0.88–0.96) and partial 64% (0.34–0.86) crowns, in favor of full crown reconstructions (*p* < 0.05). Biologic and esthetical success rates of full crowns (91% (0.79–0.97) vs. 99% (0.92–1.00)) were comparable to those of partial crowns (69% (0.42–0.87) vs. 90% (0.65–0.98)). This meta-analysis suggests that in case of possible technical failure a full crown reconstruction should be preferred compared to a partial crown.

Restoration failures are considered as such when they need repair or replacement, the general assessment of these failures can also be considered in terms of success rates. The success rates, assessed by biologic, technical and esthetical aspects showed a decrease in success from 24 to 36 months. Compared to previous reviews the present data were higher compared to ceramic, zirconia and CAD/CAM single crown reconstructions reported in previous studies [16,48,56].

This study assessed the failures as either biologic, technical and esthetic complications, although during the analysis of the included studies, the lack of homogeneity of the results did not allow for its specific analysis resulting in an overall complications analysis. Considering tooth-supported restorations complications, the success ratio of biologic complications decreased in case of caries occurrence, loss of pulp vitality, endodontic treatment, tooth fracture and hypersensitivity. The present study showed a biologic success rate of 88% at the follow-up period ≤24 m and 75% at ≥36 m. The most frequent biologic complication reported in the literature was caries and loss of pulp vitality. Comparing full and partial restorations higher biologic complications rates (21% more) were observed in partial reconstructions. Considering the characteristics of partial restorations, in terms of indications and dental preparation, full crowns could hide biologic complications. Therefore, caries can be diagnosed more easily in partial crowns compared to full crowns and could explain the results obtained in this study. The biologic complications for full crowns were lower in metal-ceramic restorations than in full ceramic reconstructions [16,57].

Technical complications include ceramic fracture, cracks, core failure, chipping, problems with microleakage and the loss of retention. Ceramic chipping has been described as the most common technical complication, finding similar ranges for metal ceramics and fully ceramic crowns with no statistic differences between materials. However, the overall technical complication rates in the present study were higher compared to conventional and other CAD/CAM materials [16,57].

Missing clinical workflows and lacking experience with these newly developed materials could have an influence in the complications derived from bonding techniques and microleakage, factors such as polymerization of resin cement, degradation of adhesive, enzymatic degradation of bonding of these materials composition could explain the higher failure rates compared to conventional groups or metal-ceramic restorations regarding biologic and technical complication rates [51].

The technical complications in partial restorations are increasing during the follow-up assessment and between groups showing less complications for full coverage restorations. Considering the design and the manufacturing process, the complications could have been due to defects of the thickness and the roughness of the final preparations milled by CAD/CAM chairside units. Some partial crowns are designed and milled using chairside devices, lacking a verification of material thickness throughout the technician. Technical complications may also result in esthetical problems, such as discoloration or wear of glace. The results of the review for esthetic were higher at 36 months and however lower compared to the other studies. Considering the posterior localization of the restorations, it is possible that the results are due to the fact that materials are biomimetic, and patients do notice esthetical failures less than in the anterior sites.

Given these data, the results for the CAD/CAM crowns of hybrid polymer and ceramics are comparable regarding the 5-year success rates performance with other materials.

A tendency for lower failure rate for glass-matrix ceramics and polycrystalline ceramics compared to leucite and feldspathic ceramic could be observed. The high survival rate of glass-matrix ceramics—followed by resin-matrix ceramics and polycrystalline ceramics—should, however, be considered with caution due to shorter follow-up periods of the latter materials.

Dual curing agents are preferred for ceramic and resin-matrix ceramic inlays in order to compensate for the light transmission throughout the restoration and to allow complete polymerization even at the bottom of the cavity, where the access of LED curing light is limited [58]. Despite the wide diversity of included materials, most studies used chemically polymerized or LED polymerized dual curing agents. In studies where chemical and dual curing cements were compared, the dual curing systems achieved better results and presented lower failure rates compared to only chemical luting agents.

According to the findings of this systematic review, a great heterogeneity of the methodological data between studies with lack of properly comparations (control and study groups), no homogeneous restoration material type groups and a short follow-up examination was observed. More homogeneous studies with the more comparable materials, manufacturing techniques and CAD/CAM software system with a control groups in a split-mouth randomized controlled study design should be conducted.

The density of published high survival rates is statistically slightly conspicuously high. In the lower section, there is the study by Baader et al. 2016, which stands out regarding the low survival ratios. However, further small studies, which published a low outcome are lacking.

## 5. Conclusions

Summary for success rates and different follow-up times including all biologic, technical and esthetical parameters could be listed as follows:

- All success rates decreased after 36 or more months compared to 24 months;
- The esthetic success rates were greatest, followed by the almost identical rate of technical and biologic success rates;
- There were no significant differences at the 95% level between the two follow-up times nor between the biologic, technical and esthetic aspects;
- Both the biologic, technical and esthetic success rates were higher for full crowns than for partial crowns;
- The technical success rate of full crowns was statistically significantly higher than that of partial crowns;
- The esthetic success rates are greater than the biologic or technical ones, but neither for the full crowns nor for the partial crowns these comparisons were of significance.

## Figures and Tables

**Table 1:** Search strategy according to the focused question (PICO).

Focused Question (PICO)	In Patients Receiving Tooth-Borne Partial or Full Crowns, Are Monolithic CAD/CAM Restorations Comparable to Conventionally Manufactured Restorations in Terms of Survival and Clinical Success Rates?	
**Search strategy**	**Population**	Tooth-borne partial or full crowns. #1—((dental crowns [MeSH]) OR (dental restoration permanent [MeSH]) OR (full crown) OR (partial crown) OR (table top))
	**Intervention**	Monolithic CAD/CAM restorations. #2—((computer-aided design [MeSH])) OR (computer-assisted design [MeSH]) OR ((computer-aided manufacturing [MeSH])) OR (computer-assisted manufacturing [MeSH]) OR (cerec [MeSH]) OR (CAD/CAM) OR (rapid prototyping)) #3—((ceramics [MeSH]) OR (dental porcelain [MeSH]) OR (polymers [MeSH]) OR (monolithic))
	**Comparison**	Conventionally manufactured restorations. #4—((porcelain-fused to metal) OR (lost-wax technique)) #5—(dental alloys [MeSH])
	**Outcome**	Survival (rates) and/or clinical success. #6—((survival analysis [MeSH Terms]) OR (survival rate [MeSH Terms]) OR (survival)) #7—((success) OR (failure) OR (dental restoration failure [MeSH Terms]) OR (complications [MeSH Terms]) OR (clinical behavior) OR (adverse event) OR (chipping) OR (debonding))
	**Search combination(s)**	(#1) AND (#2 or #3) AND (#6 or #7)

**Table 2:** Quality assessment of included studies using the Newcastle–Ottawa scale.

Study	Selection				Comparability	Outcome			Numbers of Stars (Out of 8)
	1	2	3	4	1	1	2	3	
Botto et al. 2016	–	★	–	–	★	★	★	★	5
Guess et al. 2014	★	★	★	★	★	★	★	★	8
Dhima et al. 2014	–	–	–	–	★	★	★	★	4
Dukic et al. 2010	–	–	–	–	★	★	★	★	4
Azevedo et al. 2012	★	★	★	★	★	★	★	★	8
Gehrt et al. 2013	★	★	★	★	★	★	★	★	8
Guess et al. 2014	★	★	★	★	★	★	★	★	8
Rauch et al. 2018	★	★	–	–	–	★	–	–	3
Reich et al. 2013	★	★	–	–	–	★	–	–	3
Santos et al. 2016	★	★	★	★	★	★	★	★	8
Santos et al. 2013	★	★	★	★	★	★	★	★	8
Taschner et al. 2012	★	★	★	★	★	★	★	★	8
Taskonak et al. 2006	★	★	–	★	★	★	★	–	6
Krejci et al. 1992	–	★	–	–	–	★	–	–	2

★: Each star corresponds to the subsection of quality assessment criteria.

**Table 3:** Characteristics of included studies.

Author/ Publication Year	Journal	Study Type	Patients (*N*)	Restoration (*n)*	Ratio (*n*/N)	Follow-up	Drop-out	Material	Manufacturing Technique	Luting Agent	Failure	Survival	Success	Outcome
Mittal et al. 2016 [36]	J Clin Ped Dent	RCT	50	50	1	36 Months	0	IRC (indirect resin composite) vs. SSC (stainless steel crowns)	IRX (Composite 3-M Espe) SSC	IRC (Dual cure resin cement RelyX) SSC (luting glass ionomer cement Fuji I)	IRC (3) SSC (2)	IRC (82.9%) SSC (90.7%)	IRC (100%) SSC (95%)	Modified FDI criteria’ Dental chair side treatment time and postoperative acceptability Marginal integrity IRC < SSC Time/esthetic: IRC > SSC
Botto et al. 2016 [23]	Am J Dent	Retrospective	47	93	93/47	5–18 years		13 onlays feldspathic porcelain (Vitadur Alpha), 78 onlays, 2 inlays IPS-Empress		RelyX	6 (6.5%)	87 (93.5%)	81 (93%)	Gender, age, tooth preparation, number, type, extent, location, quality and survival of the restorations, ceramic materials, luting resin cements, parafunctional habits, secondary caries and maintenance therapy, marginal adaptation, marginal discoloration, occlusal surfaces
Baader et al. 2016 [22]	J Adhes Dent	RCT	34	68	2	6.5 years	16 patients	Vita Mark II; Cerec 3D	Indirect cast	RelyX With/without enamel etching	16:11 RXU PCCs and 5 RXU+E PCCs failed. The reasons for this were fractures of restorations (3 RXU, 4 RXU+E), debonding of PCCs with no possibility of recementation (4 RXU), one endodontic treatment followed by renewal of the restoration (1 RXU) and one renewal of the PCC due to caries at another site of the tooth, necessitating a full-crown preparation (1 RXU)	RXU of 60% and for RXU+E of 82%,	–	Modified USHPS postoperative hypersensitivity, anatomic form, marginal adaptation, marginal discoloration, surface texture and recurrent caries.
Seydler et al. 2015 [44]	J Prosthet Dent	RCT	60	60	1	2 years	0	veneered zirconia (VZ) group were made of zirconia frameworks veneered with CAD/CAM-produced lithium disilicate ceramic; monolithic lithium disilicate (MLD) ceramic	MLD crowns were milled (Cerec MC XL; Sirona Dental Systems) from a block (IPS e.max CAD; Ivoclar Vivadent AG) VZ crowns were milled from a zirconia blank (IPS e.max ZirCAD; Ivoclar Vivadent AG); the veneer structure was milled from an IPS e.max CAD lithium disilicate blank (both, Cerec MC XL; Sirona Dental Systems).	(Multilink; Ivoclar Vivadent AG	none	100		USHPS The quality of marginal fit, color and technical and biologic complications were recorded.
D’all’Orologio et al. 2014 [24]	Am J Dent	RCT	50	150		8 years	30 restoration, 10 patients	100 with the new restorative material, 50 with the composite as control, XP Bond ceram.x Duo Esthet.X		bonding system (XP Bond)	7% There were eight failures in the experimental group and four failures in the control group here were two key elements of failure: the presence of sclerotic dentin and the relationship between lesion and gingival margin.	93%		Retention, Sensitivity, Marginal Integrity, Caries, Contour
Akin et al. 2014 [20]	J Prosthodont	RCT	15	30	2	2 years	0	all-ceramic crowns	fabricated with CAD/CAM and heat-pressed (HP) techniques	Variolink II/Syntac; Ivoclar Vivadent	0	100		Porcelain fracture and partial debonding that exposed the tooth structure, secondary caries, extraction of abutment teeth and impaired esthetic quality or function were the main criteria for irreparable failure.
Guess et al. 2014 [32]	Int J Proshodont	Prospective clinical study	25	86	86/25	7 years	11 patients	all-ceramic veneers with overlap (OV) and full veneer (FV) preparation designs	Leucite-reinforced glass-ceramic veneers (IPS Empress, Ivoclar Vivadent)	(Variolink II, Ivoclar Vivadent	One OV restoration fractured (Figure 2a). cohesive ceramic fracture and crack formation within the restoration material were noted in 12 patients.	100% for FV restorations and 97.6% for OV restorations.	0.85 (CI: 0.70 to 1.00) for the FV restorations and 0.70 (CI: 0.45 to 0.95) for the OV restorations	USPHS criteria
Selz et al. 2014 [43]	Clin Oral invest	RCT	60	149	>2	5 years			In-Ceram Alumina crowns	62 Panavia, 59 Super-Bond C&B; 28 Ketac	Endodontic treatment was carried out on 7.4% of all abutment teeth and 5.4% revealed secondary caries. Unacceptable ceramic fractures were observed in 7.4%. Debonding was a rare complication (1.3%).	91.6% for Super Bond C&B-, 87.4% for Ketac Cem- and 86.3% for Panavia F-bonded	82,2 Panavia, 88.7 Super-Bond C&B; 80.1 Ketac	secondary caries, clinically unacceptable fractures, root canal treatment and debonding.
Özsoy et al. 2016 [38]	JAST	RCT	60	67	>1	2 years	2 teeth	indirect composite onlays and overlays	indirect composite (Gradia, GC, Japan)	Variolink II		100		Anatomy, marginal adaptation, marginal discoloration, color match, surface roughness, caries
Dhima et al. 2014. CAVE: Tooth & implant-borne [25]	J Prosthet Dent	Retrospective	59	226	226/59	5 years		Ceramic single crown				95%		
Dukic et al. 2010 [26]	Oper Dent	Prospective study	51	71	71/51	3 years		Ind. comp	35 Ormocer, Admira, 36 Grandio	Grandio with Voco Bifix QM	0	100	No significance Ormocer/Grandio	Modified USHPS
Azevedo et al. 2012 [21]	Braz Dent J	Prospective study	25	42	42/25	1 year	0	23 etched, non-etched, 19 etched (Filtek Supreme XT; 3M ESPE)	stone dies by the incremental technique using a LED device with power density of 1000 mW/cm^2^	Etched group (ETR)—selective enamel phosphoric-acid etching + RelyX Unicem clicker; 2. Non-etched group (NER)—RelyX Unicem	0	100		More than 99% of the scores were considered clinically excellent (Alpha 1) or good (Alpha 2). Only 3 scores (0.9%) were classified as clinically sufficient (Bravo): 2 from ETR group (MS = 1, Figure 3; SE = 1) and 1 from NER group
Fasbinder et al. 2010 [28]	J Am Dent Assoc	Prospective study	43	62	62/43	2 years	1.6%	lithium disilicate (IPS e.max CAD, Ivoclar Vivadent, Amherst, N.Y.) all-ceramic crowns.	chairside computer-aided design/computer-aided manufacturing (CAD/CAM) system (CEREC 3, Sirona Dental Systems, Charlotte, N.C.) e.max CAD Crystall./Glaze paste (Ivoclar Vivadent) with shade tints	Multilink Automix, Ivoclar Vivadent OR: experimental self-adhesive, dual-curing cement (EC) developed by Ivoclar Vivadent.	0	100		Modified USHPS
Frankenberger et al. 2008 [29]	J Adhes Dent	Controlled clinical trial	34	96	96/34	12 years	40%	Leucite-reinforced glass ceramic IPS Empress	according to the manufacturer’s instructions	4 cements: Dual Cement (*n* = 9), Variolink Low (*n* = 32), Variolink Ultra (*n* = 6) and Tetric (*n* = 49) (all Ivoclar Vivadent).	16% (15/96) without dropout	58 86%		luted with dual-cured resin composites revealed significantly fewer bulk fractures Surface roughness (loss of gloss), color match (improving with time), marginal integrity (distinct deterioration with marginal fractures in two cases with charlie scores after 12 years), tooth integrity (enamel cracks, one case rated Delta), inlay integrity (continuous deterioration over time, predominantly chipping of the ceramic, two charlie and two delta scores) and hypersensitivity
Frankenberger et al. 2009 [30]	Dent Mater	RCT	39	98	98/39	4 years	3%	Cergogold glass ceramic inlays	One dental ceramist produced all inlays according to the manufacturer’s instructions and recommendations within 2 weeks after impression taking.	Multibond and Definite Ormocer resin composite Definite Multibond/Definite (*n* = 45) Syntac/Variolink Ultra (*n* = 53)	21 restorations had to be replaced due to inlay fracture (*n* = 11), tooth fracture (*n* = 4), hypersensitivities (*n* = 3) or marginal gap formation (*n* = 3).	77 survival rate 89.9%,	significantly changed over time: color match, marginal integrity, tooth integrity, inlay integrity, sensitivity, hypersensitivity and X-ray control Color match was inferior for Variolink, but only at the 2-year recall (Mann–Whitney *U-*test, *p* < 0.05), marginal integrity was inferior for Variolink, but only at the 0.5 and 1-year recall (Mann–Whitney *U-*test, *p* < 0.05) and proximal contacts were inferior in the definite group, but only at baseline	criteria marginal integrity, tooth integrity and inlay integrity
Gehrt et al. 2013 [31]	Clin Oral invest	prospective study	41	104	104/41	9 years	4 patients, 10 crowns	lithium-disilicate crowns	frameworks were laminated by a prototype of a veneering material combined with an experimental glaze. lost-wax technique	adhesively luted (69.2%) or inserted with glass–ionomer cement (30.8%). adhesively luted (IPS Ceramic etchant/Monobond S/dual-cured Variolink II, Ivoclar Vivadent) and 32 (30.8%) crowns were inserted with glass–ionomer cement (Vivaglass, Ivoclar Vivadent)	4 (4.3%)	97.4% after 5 years and 94.8% after 8 years	There were five rated technical complications (5.3%). Three crowns (3.3%) suffered from minor chipping of the veneering material. Major chippings did not occur. There were four biologic complications (4.3%). Two anterior crowns (2.1%) had to be treated endodontically 94.7 months after insertion.	Biologic complications such as loss of vitality joined by declined endodontic condition, endodontic dis- ease and occurrence of caries & Technical complications such as loss of retention, minor chipping
Guess et al. 2014 [32]	Int J Proshtodont	Prospective Study	25	80	80/25	7 years	42 restorations	40 lithium disilicate pressed PCRs (IPS e.max-Press, Ivoclar Vivadent) and 40 leucite-reinforced glass–ceramic CAD/CAM PCRs (ProCAD, Ivoclar Vivadent).	computer-aided design/computer-assisted manufacture (CAD/CAM) ProCAD, Ivoclar Vivadent; Cerec 3 InLab, Sirona	hybrid composite resin material (Tetric/Syntac Classic, Ivoclar Vivadent)	1 restoration	100% for pressed PCRs and 97% for CAD/ CAM PCR	No secondary caries, endodontic complications or postoperative complaints were ob- served. Minimal cohesive ceramic fractures (Figure 2a,b) were noted in 5 patients, but all affected restorations remained in situ 0.84 (CI: 0.70–0.98) for the pressed PCRs and 0.58 for the CAD/CAM PCRs (CI: 0.38–0.78).	modified United States Public Health Service (USPHS)
Murgueitio et al. 2012 [37]	J Prosthodont	Prospective study	99	210	210/99	3 years	?	leucite-reinforced IPS Empress Onlays and Partial Veneer Crowns	the manufacturer’s instructions using the vacuum injection mold technique for leucite-reinforced ceramic material (IPS Empress).	Variolink II, Ivoclar Vivadent	The mode of failure was classified and evaluated as (1) adhesive, (2) cohesive, (3) combined failure, (4) decementation, (5) tooth sensitivity and (6) pulpal necrosis 33%	96.66%	Increased material thickness produced less probability of failures. Vital teeth were less likely to fail than nonvital teeth. Second molars were five times more susceptible to failure than first molars. Tooth sensitivity postcementation and the type of opposing dentition were not statistically significant in this study.	USPHS
Esuivel-Ipshaw et al. 2012 [27]	J Prosthodont	RCT	32	37	37/32	3 years	1 restoration	(1) metal-ceramic crown (MC) made from a Pd–Au–Ag–Sn–In alloy (Argedent 62) and a glass- ceramic veneer (IPS d.SIGN veneer); (2) non-veneered (glazed) lithium disilicate glass–ceramic crown (LDC) (IPS e.max Press core and e.max Ceram Glaze); and (3) veneered lithia disilicate glass–ceramic crown (LDC/V) with glass–ceramic veneer (IPS Empress 2 core and IPS Eris).		Variolink II, Ivoclar Vivadent	0?	100?	between years 2 and 3, gradual roughening of the occlusal surface occurred in some of the ceramic-ceramic crowns, possibly caused by dissolution and wear of the glaze. Statistically significant differences in surface texture (*p* = 0.0013) and crown wear (*p* = 0.0078) were found at year 3 between the metal-ceramic crowns and the lithium-disilicate-based crowns.	tissue health, marginal integrity, secondary caries, proximal contact, anatomic contour, occlusion, surface texture, cracks/chips (fractures), color match, tooth sensitivity and wear (of crowns and opposing enamel). Numeric rankings ranged from 1 to 4, with 4 being excellent and 1 indicating a need for immediate replacement.
Manhart et al. 2010 [35]	Quintessence Int	RCT	89	155	155/89	3 years	Artglass inlays (35%) and Charisma inlays (21%)	Resin composite	The inlays were postcured in a light oven (Uni-XS, Heraeus Kulzer)	adhesive system Solid Bond (Heraeus Kulzer)	five Artglass and 10 Charisma inlays failed mainly because of postoperative symptoms, bulk fracture and loss of marginal integrity	5 Artglass and ten Charisma inlays had to be (3 years)	Small Charisma inlays exhibited a statistically significant better performance for the “integrity of the restoration” parameter (*p* = 0.022).	Modified USPHS
Rauch et al. 2018 [39]	Clin Oral invest	Prospective	34	41	41/34	10 years	15 restorations	monolithic lithium disilicate crowns	chairside CAD/CAM technique.	Multilink Sprint, Ivoclar Vivadent	5 five failures occurred due to one crown fracture, an abutment fracture, one endodontic problem, a root fracture and a replacement of one crown caused by a carious	24/29	Due to the small amount of technical complications and failures, the clinical performance of monolithic lithium disilicate crowns was completely satisfying.	Modified USHPS
Reich et al. 2013 [40]	Clin Oral invest	Prospective clinical trial	34	41	41/34	4 years	12 restoration	lithium disilicate crowns	chairside CAD/CAM technique (Cerec)	Multilink Sprint (Ivoclar-Vivadent)	1 failure 96.3% after 4 years according to Kaplan–Meier	28	The complication-free rate comprising all events after 4 years was 83%, whereas the rate dropped down to 71% after 4.3 years	Modified USHPS
Santos et al. 2016 [41]	Clin Oral invest	Prospective clinical trial	35	86	86/35	5 year	17.91% restoration	sintered Duceram (Dentsply Degussa) and pressable IPS Empress (Ivoclar Vivadent).	poured with dental stone type IV (Durone, Dentsply).	Variolink II, Ivoclar Vivadent	8 failures Four IPS restorations were fractured, two restorations presented secondary caries (one from IPS and one from Duceram) and two restorations showed unacceptable defects at the restoration margin and needed replacement (one restoration from each ceramic system).	56	87% significant differences in relation to marginal discoloration, marginal integrity and surface texture between the baseline and five-year recall for both systems	Modified USHPS
Schenke et al. 2012 [42]	Clin Oral invest	RCT	29	58	58/29	2 years	0	ceramic blocks (Vita 3D Master CEREC Mark II, CAD/CAM designed and machined with the CEREC III system (Sirona CEREC III Software Version 3.0 (600/800), Sirona, Bensheim, Germany)	an indirect method on a die cast	RelyX Unicem with/without enamel etching	4 failures	54	Statistically significant changes were observed for marginal adaptation (MA) and marginal discoloration (MD) between BL and 2 years, but not between the two groups (RXU, RXU+E). Percentage of alfa values at BL for MA (RXU, 97% and RXU+E, 100%) and for MD (RXU, 97% and RXU+E, 97%) decreased to RXU, 14% and RXU+E, 28% for MA and to RXU, 50% and RXU+E, 59% for MD after 24 months.	Modified USHPS
Taschner et al. 2012 [45]	Dent Mater	Prospective controlled clinical study	30	83	83/30	2 years	0	IPS-Empress	at a commercial dental laboratory according to manufacturer’s instructions	Group 1: 43 inlays/onlays were luted with RX; group 2: 40 inlays/onlays were luted with Syntac/Variolink II low viscosity (SV, Ivoclar Vivadent).	1	82/83 restorations	Indirect restorations luted with RX showed lower tooth and marginal integrity compared to the multistep approach.	Surface roughness, Color match, Anatomic form, Marginal integrity, Integrity tooth, Integrity inlay, Proximal contact, Changes in sensitivity, Radiographic check, Subjective satisfaction
Taskonak et al. 2006 [46]	Dent Mater	Prospective clinical trial	15	40	40/15	2 years		lithia-disilicate-based all-ceramic (Empress II) FDP/Crowns (20 FDPs/20 crowns)			10 (50%) catastrophic failures of FPDs occurred			marginal adaptation, color match, secondary caries and visible fractures in the restorations
Krejci et al. 1992 [34]	Quintessence Int	Prospective clinical trial	10	10	1	1.5 years	0	IPS/Empress Inlays	According to manufacturer’s instruction	Dual curing composite, Dual cement, Vivadent, Inc.	0	100	1 hypersensitivity, Discoloration at the marginal	Modified USHPS
Azevdo et al. 2012 [21]	Braz Dent J	Prospective clinical trial	25	42	42/25	1 year	0	Indirect resin composite	The composite resin restorations were built over plaster casts using the incremental technique with a LED device for light-curing the increments	1. Etched group (ETR)—selective enamel phosphoric-acid etching + RelyX Unicem clicker; 2. Non-etched group (NER)–RelyX Unicem RelyX	0	100	More than 99% of the scores were considered clinically excellent (Alpha 1) or good (Alpha 2) (Figure 2). Only 3 scores (0.9%) were classified as clinically sufficient (Bravo): 2 from ETR group (MS = 1, Figure 3; SE = 1) and 1 from NER group (SE).	Modified USHPS
